# Preserved Transcallosal Inhibition to Transcranial Magnetic Stimulation in Nondemented Elderly Patients with Leukoaraiosis

**DOI:** 10.1155/2013/351680

**Published:** 2013-07-25

**Authors:** Giuseppe Lanza, Rita Bella, Salvatore Giuffrida, Mariagiovanna Cantone, Giovanni Pennisi, Concetto Spampinato, Daniela Giordano, Giulia Malaguarnera, Alberto Raggi, Manuela Pennisi

**Affiliations:** ^1^“G. F. Ingrassia” Department, Section of Neurosciences, University of Catania, 78 Via Santa Sofia, 95123 Catania, Italy; ^2^Department of Neurology I.C., Oasi Institute (IRCCS), 73 Via Conte Ruggiero, 94018 Troina, Italy; ^3^Department of Electrical, Electronics and Informatics Engineering, University of Catania, 6 Viale Andrea Doria, 95125 Catania, Italy; ^4^Unit of Neurology, Morgagni-Pierantoni Hospital, 34 Via Carlo Forlanini, 47121 Forlì, Italy; ^5^Department of Chemistry, University of Catania, 6 Viale Andrea Doria, 95125 Catania, Italy

## Abstract

Structural corpus callosum (CC) changes in patients with leukoaraiosis (LA) are significantly associated with cognitive and motor impairment. The aim of this study is to investigate the transcallosal fibers functioning by means of transcranial magnetic stimulation (TMS) in elderly patients with LA. The resting motor threshold (rMT), the motor-evoked potentials (MEPs), and the controlateral (cSP) and ipsilateral silent periods (iSP) were determined using single-pulse TMS in 15 patients and 15 age-matched controls. The neuropsychological profile and the vascular burden at brain magnetic resonance imaging (MRI) were concomitantly explored. Patients reported abnormal scores at tests evaluating executive control functions. No significant difference was found in TMS measures of intra- and intercortical excitability. No CC lesion was evident at MRI. Transcallosal inhibitory mechanisms to TMS seem to be spared in LA patients, a finding which is in line with neuroimaging features and suggests a functional integrity of the CC despite the ischemic interruption of corticosubcortical loops implicated in cognition and behavior. The observed neurophysiological finding differs from that reported in degenerative dementia, even in the preclinical or early stage. In our group of patients, the pure extent of LA is more related to impairment of frontal lobe abilities rather than functional callosal changes.

## 1. Introduction

Several structural and functional neuroimaging studies showed that corpus callosum (CC) changes might be present in the early stages of Alzheimer's disease (AD) and even in mild cognitive impairment, supporting the crucial role of early intercortical disconnection in the dementing process [1–3]. Moreover, the disease severity seems to correlate with the degree of callosal atrophy, which is more pronounced in the latest stage of AD [4]. Similarly, the callosal tissue loss in the context of diffuse white matter lesions (WMLs) with the longitudinal magnetic resonance imaging (MRI) was significantly associated with global cognitive impairment and motor deficit, with a different rate in subjects converting to dementia compared with nonconverters [5].

Integrity of callosal fiber bundles may also be investigated electrophysiologically with transcranial magnetic stimulation (TMS) by means of the transcallosal inhibition (TI) study. TMS technique is a neurophysiological investigation allowing a safe and noninvasive assessment of motor pathways within the central nervous system. TMS applied over the primary motor cortex elicits a motor-evoked potential (MEP) in the controlateral target muscles, and it has been used to evaluate the motor threshold (MT), which is known to reflect the level of neuronal excitability [6]. The interval of suppression of the voluntary electromyographic (EMG) activity in the target muscle following a TMS stimulus on the controlateral hemisphere, the so-called controlateral silent period (cSP), is a measure of motor cortex inhibition [7]. The ipsilateral silent period (iSP), evoked by stimulating the muscle and hemisphere of the same side, is considered to reflect the interhemispheric corticocortical inhibitory mechanisms [8, 9], and it is thought to be modulated by excitatory transcallosal output neurons projecting to controlateral GABAergic inhibitory interneurons [9]. TMS analysis of the TI may be performed by measurement of the iSP.

More recently, TMS has been used in the treatment of some neuropsychiatric disorders [10–12] and to study patterns of cortical excitability in physiological and pathological brain aging [13]. In dementing processes, most TMS studies reported that motor cortex excitability is generally increased [14–19] as being probably the result of compensatory mechanisms in response to neuronal loss.

The structural CC changes with longitudinal quantitative MRI and a parallel cognitive and motor impairment were recently seen in patients with leukoaraiosis [5, 20, 21]. On the contrary, data assessing CC neurophysiological functioning level are lacking. The aim of the present study is to assess transcallosal fibers functioning in nondemented elderly patients with LA compared with age-matched controls. 

## 2. Material and Methods

### 2.1. Subjects

The study included 15 right-handed patients (mean age: 71.40 ± 5.53 years) with LA and 15 age-matched controls (mean age: 68.67 ± 3.62 years) all consecutively recruited from the database based on the Cerebrovascular Disease Center of the University of Catania (Italy). The patients did not meet the Diagnostic and Statistical Manual for Mental Disorders-Forth Edition (DSM-IV) criteria for dementia but fulfilled the brain imaging criteria for subcortical vascular disease with predominant WMLs [22]. They showed impairment in at least one cognitive domain and normal abilities in activities of daily living, thus assuming a clinical picture of vascular cognitive impairment-no dementia (VCI-ND). Controls did not have vascular lesions or other abnormal findings at brain imaging. Patients and controls were treated for their vascular risk factors with anti-platelet or anticoagulant medications (aspirin, clopidogrel, and warfarin), antihypertensive drugs (angiotensin-converting enzyme inhibitors, angiotensin II receptor antagonist, diuretics, and calcium channel blockers), cholesterol-lowering medications (statins), and oral antidiabetic drugs or insulin. None of the participants was on antidepressant or other psychotropic drugs. Patients with a history of major neurological disorders (i.e., dementia, stroke, Parkinson's disease, multiple sclerosis, and epilepsy) or major psychiatric illness, head trauma, acute or chronic noncompensated medical illness (such as heart failure, liver cirrhosis, kidney failure, respiratory failure, severe metabolic imbalance, and diffuse neoplasm), and alcohol or drug abuse were excluded. Additional exclusion criteria included mini-mental state examination (MMSE, 1975) <24, conditions precluding MRI or TMS execution, and use of drugs able to modulate cortical excitability. All participants underwent electroencephalogram to rule out predisposition to seizures. The study was approved by the local ethical committee, and written informed consent was given by all individuals. This study has been conducted in accordance with the Declaration of Helsinki (1964).

### 2.2. Assessment

All subjects underwent a clinical evaluation, including age, gender, education, presence of cerebro-cardiovascular risk factors (hypertension, diabetes, hypercholesterolemia, coronaropathy, atrial fibrillation, and smoking habit), and general and neurological exams. The neuropsychological battery of tests assessed overall cognitive impairment (MMSE), frontal lobe abilities (frontal assessment battery, FAB) [23], interference task stroop color-word test [24], depressive symptoms (the 17-item Hamilton depression rating scale), apathy (apathy scale) [25], and functional status (activity of daily living [26] and instrumental activity of daily living [27]). The brain MRI was acquired from patients and controls with a 1.5 T general electric system. The imaging protocol consisted of T1-, T2-, proton density-weighted, and fluid-attenuated inversion recovery scans, including scans on the sagittal and coronal planes for a proper visualization of CC; slice thickness was 5 mm with 0.5 mm slice gap. In the patients group, the severity of WMLs was graded according to the Fazekas visual scale [28].

### 2.3. Transcranial Magnetic Stimulation

MEPs of the right and left first dorsal interosseous (FDI) muscles were elicited using a Magstim 200 stimulator (The Magstim Company, Whitland, Dyfed) connected to a 70 mm figure-of-eight coil applied with the handle pointing backwards and laterally, at an angle of 45° to the sagittal plane, on the optimum site of stimulation which consistently yielded the largest MEP (hot spot). EMG activity was recorded from a silver/silver-chloride surface active electrode placed over the motor point of the target muscle with the reference electrode placed distally at the metacarpophalangeal joint of the index finger. Motor responses were amplified and filtered (bandwidth 3–3000 Hz) using a Medelec Synergy (Oxford Instruments) system with gains of 100 *μ*V and 5 mV/div. 

Resting MT (rMT) was defined, according to the IFCN Committee recommendation [29], as the lowest stimulus intensity able to elicit MEPs of an amplitude >50 *μ*V in at least 5 of 10 trials, with the muscle at rest. Central motor conduction time (CMCT) was calculated by subtracting the conduction time in peripheral nerves, estimated by conventional F-wave techniques, from MEP latency obtained during moderate active muscle contraction (10%–20% of maximum background force), at a stimulus intensity set at 130% of the rMT [29]. M- and F-waves were elicited by giving supramaximal electrical stimulation (constant-current square-wave pulse of 0.2 ms) to the ulnar nerve at wrist. The size of the MEPs was expressed as a percentage of supramaximal M-wave amplitude (*A* ratio). Moreover, in order to assess spinal motor excitability, the mean amplitude of the F-wave was measured in the target muscle.

The cSP and iSP were determined with an approximately 50% of maximum tonic voluntary contraction of the FDI muscles, induced by controlateral and ipsilateral single TMS pulses delivered at 130% of rMT, respectively. The mean cSP and iSP durations of 10 rectified trials were calculated. The onset latency of iSP was defined as the time interval from TMS to the decline of tonic EMG activity of more than 70% of mean EMG activity assessed over 100 ms prior to stimulation. iSP duration was measured from the onset of the previously defined decrease of tonic EMG activity to recurrence of mean prestimulus EMG activity [9].

### 2.4. Statistical Analysis

The variables obtained from patients and controls were compared using the nonparametric Mann-Whitney test for unpaired datasets and the *χ* ^2^ test for categorical variables. The Wilcoxon test for paired data was used to compare patients hemispheres. A *P* value less than 0.05 was considered statistically significant.

## 3. Results and Discussion

### 3.1. Results

Clinicodemographic and neuropsychological features of all participants are summarized in Table 1. Patients and controls were similar in terms of age, gender, educational level, and cerebrovascular risk factors profile. Soft neurological signs, such as subtle tendon reflex asymmetry, slight postural instability, and sensory disturbances, were reported in some of the patients. As expected, stroop scores were significantly higher, and FAB score was significantly lower in patients compared with controls (Table 1) although without any evidence of functional disability. At MRI, WMLs severity was graded as mild in 7, moderate in 2, and severe in 6 patients; WMLs location was widespread with a predominant frontal lobe distribution. Controls MRI was unremarkable (Fazekas 0). No evident lesion or atrophy of the CC was documented. There were no statistically significant differences for TMS measures of cortical excitability between patients and controls and between the two hemispheres (Table 2). Notably, both the iSP duration and latency did not differ between the two groups and hemispheres.

### 3.2. Discussion

The main finding of this study is the functional integrity of TI in patients with LA and cognitive profile of VCI-ND. Most of the findings assessing the role of CC changes in dementing process, especially in AD, come from neuroimaging studies, and few studies only investigated the pattern of motor cortex excitability in patients with subcortical vascular disease [30–33]. In line with the previously published data [30], we did not find change neither in the global cortical excitability nor in the intracortical inhibitory circuits, as indexed by rMT and cSP, respectively. No abnormality of interhemispheric connections in terms of TI evaluated by means of iSP was also found, supporting a relative functional CC integrity in this group of patients.

Nevertheless, WMLs of vascular origin may determine preclinical changes in motor and in nonmotor areas [34], and, with regard to CC, it is known that increasing load of age-related WMLs volume was significantly correlated with atrophy of the CC and its subregions in nondisabled elderly subjects with leukoaraiosis, suggesting that WMLs may lead to a gradual loss of CC tissue [5, 35]. CC atrophy is also significantly associated with impaired motor performance and walking speed and seems to contribute to development of dementia in the elderly independently of WMLs load [20]. However, results from other studies showed that the pure extent of WMLs may be more related to impairment of frontal lobe function rather than that of callosal atrophy [36]. Indeed, we observed worse cognitive performances in tests evaluating frontal lobe abilities despite a substantial radiological and neurophysiological CC integrity. It is likely that the preserved TI in our patients might be explained by the different mechanism involving CC dysfunction in patients with neurodegenerative dementia or with an overt disability. The impaired callosal functioning observed in AD underlies an intercortical deficit via the CC. Conversely, in our patients with an executive dysfunction without impairment of global cognitive status, a normal functioning of transcallosal inhibitory mechanisms is in line with the MRI lack of a significant CC pathology. It can be hypothesized that CC would be spared in nondemented LA patients where the intrahemispheric changes due to deep WMLs may be related to executive dysfunction and the transhemispheric connections might possibly take place via the anterior and posterior commissures. Recent data derived from the LADIS study of longitudinal quantitative MRI on a large cohort of elderly patients with age-related WMLs and no or mild impairment in instrumental activity of daily living [21] seem to support this view. The authors showed that the rates of tissue loss in the total CC area and in rostrum/genu and midbody subregions were significantly associated with decline in a compound measure of cognitive speed and motor control, but not in those of executive functions, memory, or global cognitive function. They concluded that the consequence of CC tissue loss on psychomotor function may be driven by altered interhemispheric information transfer between homologous cortical areas [21]. Alternatively, it has been postulated that, in patients with extensive LA, atrophy and reduced diffusion anisotropy of the CC may indicate diffuse hemispheric deep white matter tract damage, which may explain global cognitive impairment and development of vascular dementia [37]. Finally, cerebral microangiopathy may also affect callosal pathways by chronic demyelination [38]. 

On the contrary, in degenerative dementia, comprehensive but still subclinical dysfunctions of motor cortical inhibition with strong interactions of intra- and interhemispheric inhibitory phenomena were recently described in terms of significant prolongation of the iSP duration in mild to moderate AD patients compared with controls [39].

The main limitation of this study is the lack of a quantitative assessment of CC at conventional MRI. Recently, a combined study using diffusion tensor imaging (DTI) and TMS in AD [40] correlated the iSP and the rMT with fractional anisotropy and mean diffusivity values of the CC and corticospinal tract. Although both TMS and DTI metrics were prominently altered in AD patients, impaired white matter integrity was not associated with increased iSP latency or reduced rMT, and, therefore, beside the direct degeneration of the underlying fiber tracts, other pathophysiological mechanisms may account for the observation of decreased TI and increased motor excitability in AD.

## 4. Conclusions

Unlike dementing patients, individuals with VCI-ND seem to exhibit a functional integrity of transcallosal connectivity to TMS, which is in line with the MRI CC findings. Although this result needs to be interpreted in the context of clinical, neuropsychological, and neuroimaging data, specific electrophysiological measures might be associated to cognitive decline in the elderly, confirming the role of TMS as a powerful tool to investigate noninvasively transcallosal pathways in humans and giving new insights into the pathophysiological mechanisms underlying dementing process. Further investigations and longitudinal studies are needed to clarify the role of inter-hemispheric connections in patients with VCI-ND and the clinical relevance of CC changes in the development and progression of vascular and degenerative dementias.

## Figures and Tables

**Table 1:** Clinicodemographic characteristics and neuropsychological test scores of patients and controls.

	Patients	Controls	*P* value
Age (years)	71.40 ± 5.53	68.67 ± 3.62	NS
Gender (males/females)	9/6	9/6	NS
Educational level	7.93 ± 4.72	8.93 ± 4.83	NS
Hypertension	13 (86.67%)	10 (66.67%)	NS
Atrial fibrillation	2 (13.33%)	0 (0%)	NS
Coronaropathy	2 (13.33%)	0 (0%)	NS
Hypercholesterolemia	9 (60%)	5 (33.33%)	NS
Diabetes	8 (53.33%)	3 (20%)	NS
Smoking habit	8 (53.33%)	5 (33.30%)	NS
Neurological signs	9 (60%)	0 (0%)	**<0.01**
MMSE	27.00 ± 1.80	28.20 ± 1.73	NS
ADL	5.93 ± 0.25	6.00 ± 0	NS
IADL	7.60 ± 1.055	7.86 ± 0.35	NS
Stroop *T*	44.68 ± 16.98	27.72 ± 9.87	**<0.01**
Stroop *E*	3.74 ± 3.62	1.58 ± 2.03	**0.02**
FAB	13.64 ± 2.63	16.62 ± 1.61	**<0.01**
HDRS	5.40 ± 2.72	4.16 ± 2.32	NS
Apathy scale	0.58 ± 0.49	0.38 ± 0.34	NS

Age, education level, and neuropsychological test scores are expressed as mean ± SD.

MMSE: mini-mental state examination; ADL: activity of daily living; IADL: instrumental activity of daily living; Stroop *T*: stroop color-word test interference (time, sec); Stroop *E*: stroop color-word test interference (number of errors); FAB: frontal assessment battery; HDRS: the 17-item Hamilton depression rating scale. Statistically significant differences for *P* values are in bold.

**Table 2:** Single-pulse TMS data from both hemispheres of patients and controls.

	Left hemisphere			Right hemisphere		
	Patients	Controls	*P* value	Patients	Controls	*P* value
rMT (%)	44.60 ± 9.41	46.87 ± 6.82	NS	42.47 ± 6.54	47.13 ± 6.26	NS
cSP (ms)	81.07 ± 30.55	70.93 ± 26.99	NS	90.53 ± 39.17	73.93 ± 29.64	NS
iSP latency (ms)	44.26 ± 4.81	45.26 ± 3.01	NS	46.00 ± 4.85	44.13 ± 5.89	NS
iSP duration (ms)	11.07 ± 6.54	9.00 ± 5.17	NS	8.87 ± 7.38	6.33 ± 6.66	NS
MEP latency (ms)	21.15 ± 1.94	20.66 ± 1.51	NS	20.84 ± 2.02	20.00 ± 1.63	NS
CMCT (ms)	5.81 ± 1.07	6.29 ± 1.25	NS	5.71 ± 0.47	5.76 ± 1.20	NS
CMCTF (ms)	5.93 ± 1.17	6.02 ± 1.14	NS	5.64 ± 0.97	5.31 ± 1.15	NS
MEP *A* ratio	0.39 ± 0.12	0.33 ± 0.09	NS	0.43 ± 0.13	0.46 ± 0.12	NS
F-wave *A* (mV)	0.10 ± 0.04	0.11 ± 0.05	NS	0.13 ± 0.10	0.09 ± 0.04	NS

All values are expressed as mean ± SD. rMT: resting motor threshold; cSP: controlateral silent period; iSP: ipsilateral silent period; MEP *A* ratio: motor-evoked potential amplitude ratio; CMCT: central motor conduction time; CMCTF: central motor conduction time obtained with the F-wave; F-wave *A*: F-wave amplitude; NS: not significant.
